# Transformative learning through communal documentary viewing: a mixed methods study on kidney transplantation and organ donation in medical education

**DOI:** 10.1136/bmjopen-2024-095404

**Published:** 2025-09-28

**Authors:** Davog McCaffrey, Michael Corr, Ruth Fergie, Aisling Courtney, Tim Brown, Gerard Gormley

**Affiliations:** 1 Clinical Skills Education Centre (CSEC, Medicine), Centre for Medical Education, Queen’s University Belfast School of Medicine, Dentistry and Biomedical Sciences, Belfast, UK; 2 Regional Nephrology and Transplant Unit, Belfast City Hospital, Belfast, UK; 3 Centre for Public Health, Queen’s University Belfast School of Medicine, Dentistry and Biomedical Sciences, Belfast, UK

**Keywords:** Education, Medical, MEDICAL EDUCATION & TRAINING, Renal transplantation, NEPHROLOGY

## Abstract

**Abstract:**

**Objectives:**

To explore how learner transformation manifests in a communal arts and humanities (AH) educational activity for medical students.

**Design:**

Mixed methods explorative study.

**Setting:**

UK-based medical school that follows a 5-year case-based learning curricular model.

**Interventions:**

A group of 105 first-year medical students attended a group viewing of a TV documentary titled, ‘Life on the List’ as part of their core curriculum. The documentary explores the humanistic aspects of kidney disease, organ donation and transplantation by portraying the personal stories of transplant recipients, donors and healthcare professionals. Following the screening, attendees engaged in a question-and-answer session with an expert panel.

**Main outcome measures:**

Perceived transformation of learning was measured using a quantitative Likert-scale paired pre-screening and post-screening questionnaire. Additionally, the qualitative study used facilitated focus groups (FGs) to explore how learners may or may not have transformed their learning based on the AH educational activity.

**Participants:**

Inclusion criteria were: (a) first-year medical students and (b) those who attended the screening. Those not meeting these criteria were excluded. The quantitative questionnaire was completed by 94 participants, while 19 attended FG interviews.

**Results:**

Paired t-tests were conducted to compare pre-screening and post-screening responses across five questions. All results demonstrated statistical significance (p<0.01), reflecting perceived transformation. Using a constructivist approach and drawing on aspects of ‘Transformative Learning Theory,’ we identified five key themes regarding *how* learning was transformed: (1) an absorbing experience; (2) confronting and challenging: *different ways of seeing the world*; (3) collective reflection: *the power and safety of the crowd*; (4) ‘everything just came into perspective’: *accepting the world in a new way*; and (5) willing to change the world: *advocacy and agency*. By this collective experience focusing on real patient stories and providing an opportunity for discussion and reflection, participants were offered a holistic view on kidney medicine and transplantation. Overwhelmingly, participants were inspired to strive for person-centred care, with many being motivated to explore kidney medicine and transplantation as career options.

**Conclusion:**

Couched in pedagogy, an AH educational activity for medical students can leverage learner transformation and promote person-centred care. With respect to kidney medicine, organ donation and transplantation, such activities can provide early career exposure to these fields. Students may be inspired to act as positive ambassadors for organ donation and transplantation and further explore these areas as future career options. Further research should investigate AH interventions in diverse groups from a longitudinal perspective to consider long-term effects in promoting holistic and empathetic patient care.

**STRENGTHS AND LIMITATIONS OF THIS STUDY**

- Quantitative component: allowed us to demonstrate that perceived transformation of learning occurred following an arts and humanities (AH) educational activity.
- Qualitative component: allowed us to explore *how* learners transformed their learning based on the AH educational activity.
- Our qualitative analysis was theoretically informed (ie, using Transformative Learning Theory).
- Participants were exclusively first-year medical students at a single UK-based medical school, meaning our findings may not be applicable to other groups of healthcare professionals or different educational contexts.

## Introduction

 Arts and humanities (AH) mediums such as moving images, poetry and art have the potential to enrich the professional development of medical students, foster empathic skills and nurture person-centredness.^1^,^3^ AH interventions can be used in medical education to explore more complex and multilayered dimensions of illness experiences that more traditional methods of teaching, such as lectures or online learning, may not fully capture. These interventions aim to provide an emotive and engaging window into the life worlds of others and have previously been used in areas such as palliative medicine, clinical pharmacology and psychiatry.^4^,^8^

Integrating AH into the medical curriculum is not without challenges.^1 9 10^ In a critical narrative review, Wangding *et al* found there is a risk of such activities having greater focus on the *content* rather than the *pedagogy* needed to leverage learning from the experience.^11^ Preconceived opinions on their effectiveness can limit AH adoption in undergraduate medical curricula despite their ability to captivate the attention of learners and promote an empathic approach towards the humanistic aspects of healthcare.^12^ Therefore, insights into how best to leverage learning from AH experiences are crucial to optimise their use in healthcare education. Additionally, the ability of AH to influence important aspects of education such as role-modelling and career development remains an underexplored area within the field.

Organ donation and transplantation are areas of medicine where it is of particular importance to develop person-centred doctors.^13 14^ The profound and unique nature of transplantation for both donors (living or deceased) and recipients of these organs requires holistic and empathetic doctors to navigate this complex and emotionally challenging area of healthcare. However, in ever-competitive medical school curricula, early exposure to nephrology remains low and kidney disease often has a lower profile than that of other chronic diseases such as diabetes mellitus, heart disease or stroke.^15^ The implications of this can be seen worldwide with challenges in recruitment into both nephrology and transplantation-based specialties.^16 17^ With transplantation providing emotive patient testimonials, could an AH-based education initiative help prompt medical students to consider careers in the field?

Therefore, within the context of kidney medicine, organ donation and transplantation, we set out to address the following research question: how does transformation manifest in a communal AH educational activity for medical students?

## Methods

### Study design

This was a mixed methods explorative study. We used a quantitative paired pre-intervention and post-intervention questionnaire to determine if perceived transformation of learning occurred following an AH learning experience. We also used qualitative methods with focus groups (FGs) to explore *how* learners may or may not have transformed their learning.

### Setting

The study was conducted at a UK-based medical school that follows a 5-year case-based learning curricular model. At the end of the first year, students participate in a 'progress towards practice' development week designed to enhance readiness for more clinically oriented learning during the rest of the curriculum.

### Arts and humanities educational intervention

The AH intervention we used was a 45 min TV documentary 'Life on the List' (produced by Imagine Media Productions for BBC Northern Ireland).^18^ It was screened in a lecture theatre for 105 first-year medical students as part of their core curriculum during their development week. The documentary portrays personal stories of kidney transplant recipients, donors and healthcare professionals at different stages of the kidney transplant journey. It is important for medical students to not only understand the science that underpins kidney transplantation but also the human side of this *‘gift of life.’*

As previously highlighted by De Souza and Adams, in medical education, moving images need to be framed in a pedagogical context; it is not sufficient to merely watch a documentary.^19^ Therefore, the session was introduced by highlighting key learning outcomes regarding empathic skills development and understanding the lived experiences of patients who have kidney disease and have undergone transplantation.

Additionally, after viewing the documentary, students had the opportunity to share reflections with each other in small groups and engaged in a question-and-answer session with a panel comprising patients, caregivers and healthcare professionals specialising in kidney transplantation.

### Recruitment and sampling

All first-year medical student attendees were invited to complete the quantitative questionnaire. The purpose of the questionnaire in relation to this study was explained to all attendees via email in advance of the viewing and again at the beginning of the event. Participation was voluntary; those who did not wish to participate simply did not complete the questionnaire.

For recruitment to FGs, an email invitation with an attached participant information leaflet was sent to all first-year medical student attendees prior to the screening. Participants were selected on a first-come, first-served basis. Recruitment continued until we determined that we had achieved sufficient information power to address our research question. As is typical in qualitative research, sample sizes tend to be small to facilitate deep engagement with the data, enabling the development of themes that offer a rich and nuanced description.^20 21^

### Quantitative component of study

A paired pre-screening and post-screening ‘Likert-scale’ questionnaire was used to evaluate the opinions and perceptions of participants towards nephrology and transplantation prior to watching the documentary and immediately after watching it online supplemental appendix 1A,B.

Statistical analysis was performed using R Studio, Boston, Massachusetts, USA (V.4.3.2)^22^ to examine differences between responses to the pre-screening and post-screening questionnaire. Given the reported literature on statistical analysis of Likert responses,^23 24^ parametric testing via paired t-tests was used despite the lack of normality of the data.

### Qualitative component of study

#### Conceptual orientation

In the qualitative component of this mixed methods study, we adopted a constructivist approach, drawing on aspects of Transformative Learning Theory (TLT) and following the COnsolidated criteria for REporting Qualitative research checklist.^25 26^ Theorised by Mezirow, TLT examines how learners experience a shift in their worldview (a worldview comprises attitudes, values, narratives and expectations regarding the world, shaping our thoughts and actions)^27^ and undergo transformation through this process.^1 25^ TLT suggests that learners are transformed when they encounter a ‘disorienting dilemma.’^25^ Confronting learners in this manner and challenging their beliefs has the potential for transformative learning to be nurtured. Through a process of self-awareness and reflection, learners become open to their responses to the ‘disorienting dilemma’ and begin to critically reflect and question their worldviews.^25^ In so doing, they can gain a recognition that others may also face such dilemmas and have shared experiences. This process allows learners to reframe assumptions and consider adopting new worldviews. For this to be transformative, learners need to consider new lines of action and be willing to enact change. TLT provides a valuable lens for guiding our analysis.

#### Data collection

Four FGs were conducted by GG, DMcC, MC and RF with a total of 19 participants. The FGs were held immediately after the screening, within the medical school. Informed written consent was obtained from all participants in advance. All interviewers had been trained in qualitative methods of research and had no prior relationship with the participants. Prior to conducting the FG interviews, all interviewers met to ensure consistency in their approach. An FG interview guide was prepared, although interviewers were also encouraged to be curious and facilitate emerging discussions. Interviews were recorded using a digital Dictaphone and transcribed verbatim. Transcripts were checked for accuracy and anonymised.

#### Qualitative analysis

Consistent with our conceptual framework, we used an iterative approach to analyse our data.^25^ NVivo qualitative analysis software, Melbourne, Australia (V.12.7.0) aided in organising and categorising data.^28^ Initially, team members DMcC and GG independently immersed themselves in two contrasting FG transcripts. Using TLT principles, they established initial codes. These codes were then reviewed with other team members to discuss initial impressions and consider how assumptions and the TLT conceptual model influenced interpretations. Tentative themes were constructed, which were refined through coding additional transcripts. Researchers met regularly to share analyses, conducting comparative analyses across all transcripts until achieving theoretical sufficiency and consensus on research themes.

#### Reflexivity

During the study, the research team were continually reflexive and journaled their discussions. They held regular meetings and conversations about their assumptions and the potential for these to influence the analytical process. There was a shared understanding that having such a diverse research team could enrich the analytical process.

### Ethical considerations

Ethical approval was provided by the Faculty of Medicine, Health and Life Sciences Research Ethics Committee at Queen’s University Belfast (MHLS 24_78).

### Patient and public involvement

Patients and the public were involved from the beginning of this research project. The TV documentary used in this study involved real patients telling their personal stories related to kidney medicine and transplantation. A number of these individuals were involved in the inception of this research project and were kept actively involved and updated as the study progressed. As patients with lived experience and passionate advocates for organ donation and transplantation, these individuals informed the development of our research question, outcome measures and study design by considering the transformation of learning in the context of future doctors caring for patients with kidney disease and transplantation.

The findings of the study were shared with our patient and public representatives to ensure these aligned with their perspectives and experiences in healthcare, as mediated through medical education and the intervention used in this study.

## Results

### Quantitative results

There were 105 first-year medical students who attended the event and were eligible to participate in the questionnaire. Three of the 97 questionnaires received were excluded due to incomplete responses, hence the complete response rate was 94/105 (89.5%). The results across five key Likert-scale questions with ratings 1–10 pre-AH and post-AH educational intervention can be seen in table 1.

**Table 1:** Pre-screening and post-screening questionnaire results and analysis (n=94)

Question	Pre-mean	Post-mean	Mean difference	P value
1. Do you feel that collective non-fictional documentary viewing is a useful tool to facilitate learning in medical education? (1=not useful at all, 10=extremely useful)	7.35	9.28	1.92	<0.01
2. Please rate your general awareness of kidney disease and transplantation. (1=no awareness, 10=unlimited awareness)	4.49	7.8	3.3	<0.01
3. How important do you feel managing people with kidney disease and transplantation will be in your future career? (1=not important at all, 10=extremely important)	7.68	8.66	0.97	<0.01
4. How confident do you feel in discussing the topic of organ donation with your future patients? (1=not confident at all, 10=extremely confident)	5.18	7.7	2.52	<0.01
5. How likely are you to consider a future career involving kidney disease and transplantation? (1=never going to consider; 10=extremely likely to consider)	5.67	8	2.33	<0.01

After watching the documentary, the perceived usefulness of documentary viewing for learning in medical education significantly increased (mean difference=1.92, p<0.01). The general awareness of participants regarding kidney disease and transplantation also improved, with pre-test and post-test mean scores of 4.49 and 7.80, respectively, (p<0.01). Prior to watching the documentary, participants demonstrated a reasonable level of appreciation of the importance of managing patients with kidney disease and transplantation in their future careers with a mean score of 7.68. Following the documentary, this increased further to a mean score of 8.66 (p<0.01). Moreover, the confidence of participants in discussing organ donation with future patients saw a notable increase (mean difference=2.52, p<0.01). After viewing the documentary, participants reported increased likelihood of considering a career involving kidney disease and transplantation, with pre-test and post-test mean scores of 5.67 and 8.00, respectively, (p<0.01).

### Qualitative results

Table 2 summarises the parameters of the four FGs.

**Table 2:** Details of focus groups

Focus group	Number of participants (P)	Male:female ratio of participants	Duration of focus group (mins:secs)
1	5	0:5	35:43
2	6	2:4	31:37
3	4	1:3	27:07
4	4	2:2	31:34

Our analysis identified five inter-related themes of how a communal AH educational intervention transformed the understanding of medical students regarding the human aspects of kidney disease, transplantation and their development as future doctors. Below is a detailed description of these themes, together with quotes from participants to bring the themes to life.

### An absorbing experience

Universally, participants voiced that watching the TV documentary communally was a highly engaging experience. The nature of the teaching medium, that is, moving images, helped participants immerse themselves. As explained by one participant:

I think because people our age like watching TV shows, like watching movies, the fact that it was like nearly you were in the cinema, I did find it, like as you say, quite engaging… I enjoyed it. (FG1, P5)

In the context of medical education, such a medium is not a traditional teaching method. This contrasting approach captured the attention of participants. However, it was acknowledged that not all teaching should, or could, be like this. As exemplified by this participant:

It is a very good tool to just spark an interest…but actually using it as one of the only ways of teaching would be very difficult, because not everyone would be able to relate to this form of teaching, and people might prefer lectures, or tutorials, over something like this. (FG1, P1)

The content of the documentary was a strong driver for participant engagement. In addition to introducing the processes and theory behind kidney transplantation, the crafted narrative and imagery in this documentary could, for some, evoke an emotional reaction, often focusing on the humanistic aspects of illness. Though a challenging subject matter, the documentary was largely received positively as a worthwhile and emotive challenge. Participants were able to relate to the stories portrayed in the documentary and deepen their connection with the human experiences depicted:

I thought the documentary itself was very moving, like even watching it. I sat beside xxx while we were watching it, and there was (sic) parts where we were both brought to tears. (FG1, P5)

The engagement of participants was enhanced by the collective nature of watching the documentary. Having a dedicated (curricular) session mediated a shared goal in the teaching experience. Compared with, for example, watching a video online in isolation, the collective experience afforded participants dedicated time to immerse themselves in the documentary; being less distracted and focusing their attention on the documentary. As one participant explained:

At the start of the year a lot of us didn’t know each other so I think there was a nice social aspect of it… you’re all in medicine for a common goal… and I suppose it just brought you together in that sense. I think the fact it was uninterrupted, free from distractions…it deserves uninterrupted time by actually being here in person, you’re actually able to give it that rather than lying in bed in your pyjamas. (FG4, P3)

Experiencing how others reacted to the documentary, often in the moment, appeared to deepen the engagement of participants. From this position, they seemed to be more open to the human experiences of illness, fostering a more embodied connection. As exemplified by this participant:

I think you’re more empathetic when you watch it with other people, because when you’re on your own… you’re less likely to be emotional about a subject, and when someone next to you is reacting to it, it kind of makes you react to it as well. It’s like seeing someone else emotional that it helps you become more empathetic towards it. (FG1, P3)

#### Confronting and challenging: different ways of seeing the world

Once absorbed in the experience of collectively watching the TV documentary, participants articulated how their assumptions were challenged in a constructive way. The TV documentary portrayed true-to-life stories. From the perspective of participants, their training often had a greater biomedical and pre-clinical focus at this stage of their studies. However, the documentary allowed participants to see how their pre-clinical knowledge can translate into clinical practice and holistic care. As articulated by this participant:

You’re put in the shoes of the patient, and you can see it from their perspective… I thought that was very valuable patient-centred care. (FG4, P1)

In keeping with TLT, this ‘confronting’ experience afforded participants different experiences from what they were used to.^2 25^ The documentary enabled participants to experience the ‘back stories’ from the lives of patients and the importance of these—in essence, these back stories became the main stories more so than the technical aspects of clinical care. This provided a new vantage point from their role of being doctors in training and the humanistic aspects of patient care. As one participant described:

I didn’t realise those little cuts between completely medical environments to completely random ones that sort of felt a sort of shock to me at first, but then when you sort of see the story play in real time… it makes it really emotional, because then you realise how abrupt it can be in a patient’s life, being completely normal, and then suddenly you’re forced into being in a bed three times a week (on haemodialysis). It just sort of really hit me completely. (FG1, P2)

Amidst an often negative portrayal of the healthcare system in the media, participants were confronted with the positive message of the ‘*gift of life*’ associated with organ donation and transplantation. Participants were inspired by the healthcare professionals involved in the documentary and viewed them as role models for their onward professional development:

Yeah, and my social media is just full of, ‘why I quit my job as a doctor’ and ‘why you shouldn’t go into medicine.’ So, I was put off for a while, like this year I was like maybe I should leave but then seeing doctors that are passionate and still love what they’re doing does really inspire me to stay so I think we need more of that. (FG3, P2)

Another confronting experience was the assumptions participants had ahead of the documentary-based educational activity. Often, participants voiced preconceived notions about the benefit of watching a documentary:

I didn’t think I was going to learn anything from it, but I definitely did. (FG 1, P3)

More often, experiencing the documentary confronted this assumption, and participants were surprised by the engagement and learning they gained.

#### Collective reflection: the power and safety of the crowd

During and following the viewing of the documentary, participants narrated how this stimulated them to critically reflect on the experience. Universally, participants expressed the importance of the social and collective dimension in helping to process and internalise the experience. During the documentary, there were many key and emotive *moments*. Such *moments* were often related to the human side of illness and could trigger participants to converse with each other. These micro-conversations enabled participants to question and reflect on what they had just experienced. As described by one participant:

The girl sitting beside me, she spoke about her family’s experience with organ donation. And then the girl behind her was saying, “oh yeah, I think I’d really like to go into renal medicine.” So, it’s facilitating a lot of these conversations that I think are very important. (FG4, P4)

The panel discussion following the documentary was particularly noteworthy. Some participants were inspired by the documentary, which empowered them to ask questions openly. Others felt less inclined to speak, citing social challenges in front of peers. However, the documentary created a sense of community and shared learning. This communal interest encouraged questions and enriched personal reflections. Many found the discussion a psychologically safe space to explore and reflect further on the subject matter. As one participant described:

Even if you’re not actively asking questions… I nearly appreciate that someone else has thought of it and asked it, just because I haven’t necessarily thought it doesn’t mean I don’t want the answer. So, it was nice doing it, not in a huge, like a bigger group… You go away knowing more information than you wouldn’t have even thought to have asked. (FG2, P2)

#### ‘Everything just came into perspective’: accepting the world in a new way

After a process of critically reflecting on the experience, participants shared what impact this had on them, both on a personal and professional level. As theorised in TLT, participants also articulated how they began to accept new perspectives on patient care.^3 25^ These primarily focused on the humanistic aspects of illness. As described by one participant:

I think the documentary itself gave me a good perspective… just seeing those patients on their journey… That was totally eye opening… if you treat the patient before you treat the disease, you can’t go too far wrong. (FG1, P5)

The experience seemed to reinforce and extend their views on the person-centred aspects of healthcare. Participants described how these unique insights harnessed feelings of empathy and kindness which they hope to apply in their future practice, as this participant exemplified:

Just like how important it is to listen to the patient and always be mindful of them when making any decisions, and always listen to what they think and what their family thinks as well is really important. (FG3, P3)

The fact that there was a collective interest in the topic contributed to a shared sense of this being an important attribute of a compassionate doctor of the future.

#### Willing to change the world: advocacy and agency

Ultimately, engaging in the documentary-based activity offered participants a new perspective on becoming more holistic doctors. Professionally, participants appeared more motivated to deliver person-centred care. Many felt this experience instilled in them a readiness to advocate for patient care as integral to their future identity as a doctor. As one participant put it:

I think the way it’s pushed me is to be more concerned with the patient themselves and have a more holistic approach. (FG2, P1)

Participants expressed feeling energised by the positive and life-changing message of organ donation. While many may be on the organ donation register themselves, they were inspired to be ‘ambassadors for change’ by opening discussions and promoting organ donation in both their personal and professional lives. As typified by this participant:

I suppose that’s the really good thing about the organ donation topic in general is viewing this with a crowd I think then facilitates these conversations and it opens up these difficult conversations about organ donation which is the real aim of any donation campaign. They’re always like—have the conversation today, don’t wait. It’s forcing you to talk about it. (FG4, P4)

Interestingly, the experience appeared to influence many participants to consider these disciplines as future careers for themselves. As this participant explained:

It seems very dynamic, and there’s just so much to it… I feel like the transformative effect that it can have on patients is something I’d really enjoy. (FG1, P1)

## Discussion

Findings from our research extend our understanding of how a communal AH educational activity can have a transformative impact on learners. Within a pedagogical framework, such an activity can help learners bridge biomedical learning and stimulate understanding of the humanistic aspects of empathetic, person-centred care. By providing crafted life stories, focusing in this instance on kidney disease, organ donation and transplantation, we can capture the attention of learners and raise consciousness of new perspectives on the human, social and emotional aspects of healthcare. From this position, they can be encouraged to align with these new perspectives, fostering change in their professional development. These findings resonate with established knowledge of AH interventions in health profession education.^1^,^12^ Our study adds to this by highlighting the importance of the pedagogical approach in leveraging transformation from such interventions.^19^

Informed by TLT, our study sheds important light on how learning manifests in such an AH intervention.^25^ First, as indicated by our findings, the context of the AH intervention must align to the needs of learners. As with all educational interventions, intended learning outcomes of the intervention need to be integrated with curricular requirements.^4 19^ Second, the choice of medium is crucial. Capturing the attention of learners is essential for effective learning. AH mediums possess an inherent ability to engage. In our study, learners engaged in a multisensory experience through a crafted documentary featuring real-life stories. Beyond sensory immersion, it’s vital that learners can emotionally connect with these experiences. Personal narratives, especially those of actual patients, enable learners to invest in the experience and remain open to their insights.^4 10^

Once immersed and captivated by this experience, learners are often confronted with new perspectives. In these emotive moments, learners can be stimulated to reflect and trigger a state of reconsidering their previously held assumptions. As evidenced in our study, learners reconsidered their established knowledge about patients who have experienced kidney disease and transplantation, which typically focused on the biomedical aspects of such care. The TV documentary helped challenge learners to extend their appreciation of the personal aspects and life stories behind these conditions and treatments.

Learning is considered by many to be a social activity, and this was confirmed in our research.^29^ In educational activities, creating space for learners to discuss and reflect on their experiences is crucial. This social learning environment in our study provided a strong foundation for learners to reflect, internalise and aspire to influence their future professional roles. Facilitated discussions fostered a psychologically safe space where students could openly explore holistic patient care, including sensitive topics like organ donation. These discussions also highlighted socioprofessional norms that serve as models for the professional growth of students. Overall, there was a collective commitment to enhance empathetic, person-centred care.

Beyond these goals, our study revealed further outcomes: the AH experience shaped the professional identities of students, career aspirations and personal motivations for social change, particularly in relation to organ donation and transplantation. This reveals the potential for AH interventions in career signposting and role-modelling. As demonstrated by our results, smaller clinical specialties, such as nephrology, could find AH interventions beneficial to maximise early exposure and interest among medical students, which may assist future workforce challenges.

As articulated by participants in this study, while AH educational interventions can be welcomed, they need to be considered in the wider context of curricular endeavours.^1 12 19^ Other methods of teaching are still required and needed (eg, lectures and work-based learning). AH educational activities can enrich such traditional teaching methods in a blended approach.

### Strengths and weakness of study

Our study possessed several strengths. First, we used a widely accessible AH intervention—a TV documentary. This format is easily adaptable for use in various educational and professional settings. Second, our study focused on the emotionally compelling subject of kidney disease, organ donation and transplantation. Providing direct and protected exposure to these topics for large cohorts of students can be challenging, but the documentary format offered a practical, relatable and immersive learning experience. Third, our methodology was grounded in a theoretical framework (TLT),^25^ which guided our approach and analysis, which can enable the transferability of our findings to different contexts.

Despite its strengths, our study has several limitations to consider. First, we focused exclusively on junior medical students at one institution using a TV documentary as our intervention. Therefore, our findings may not be applicable to other groups of healthcare professionals or different educational contexts. Second, while we explored the perceived impact on learners, we did not measure actual changes in their development or ultimate career choices, which may change significantly throughout the remainder of their medical studies. Future research should investigate a range of AH interventions in diverse groups, including senior medical students and practising clinicians. Additionally, longitudinal studies are needed to assess the long-term effects of such experiences on professional development and patient outcomes, particularly in terms of promoting holistic and person-centred care.

## Conclusion

Our research offers new insights into how learner transformation can manifest using an AH educational activity. When couched in a pedagogical framework, such activities can transform learning by encouraging learners to adopt new vantage points in their personal and professional development. Learning is a social endeavour, and AH educational interventions offer a protected time to collectively experience and reflect on an emotive topic such as kidney disease, organ donation and transplantation. Affording learners a communal experience and a safe space to promote dialogue and envisage change can be a powerful driver in enhancing learning and stimulating lines of action in their future practice.

In our study, inspired by real patient stories, participants reflected on how the ‘*gift of life*’ can transform the lives of patients and their loved ones. Participants were inspired by this life-giving message of person-centred and holistic care, with many being motivated to promote organ donation and encouraged to explore kidney medicine and transplantation as future career options.

## Supplementary material

10.1136/bmjopen-2024-095404 online supplemental file 1

## Data Availability

Data are available upon reasonable request.
