# Supplementary material for: Transformative learning through communal documentary viewing: a mixed methods study on kidney transplantation and organ donation in medical education
Source: BMJ Open. 2025 Sep 28;15(9):e095404. doi: 10.1136/bmjopen-2024-095404 (PMC12481357; doi:10.1136/bmjopen-2024-095404)

**SUPPLEMENTARY MATERIAL**

**Appendix 1a – pre-screening questionnaire**


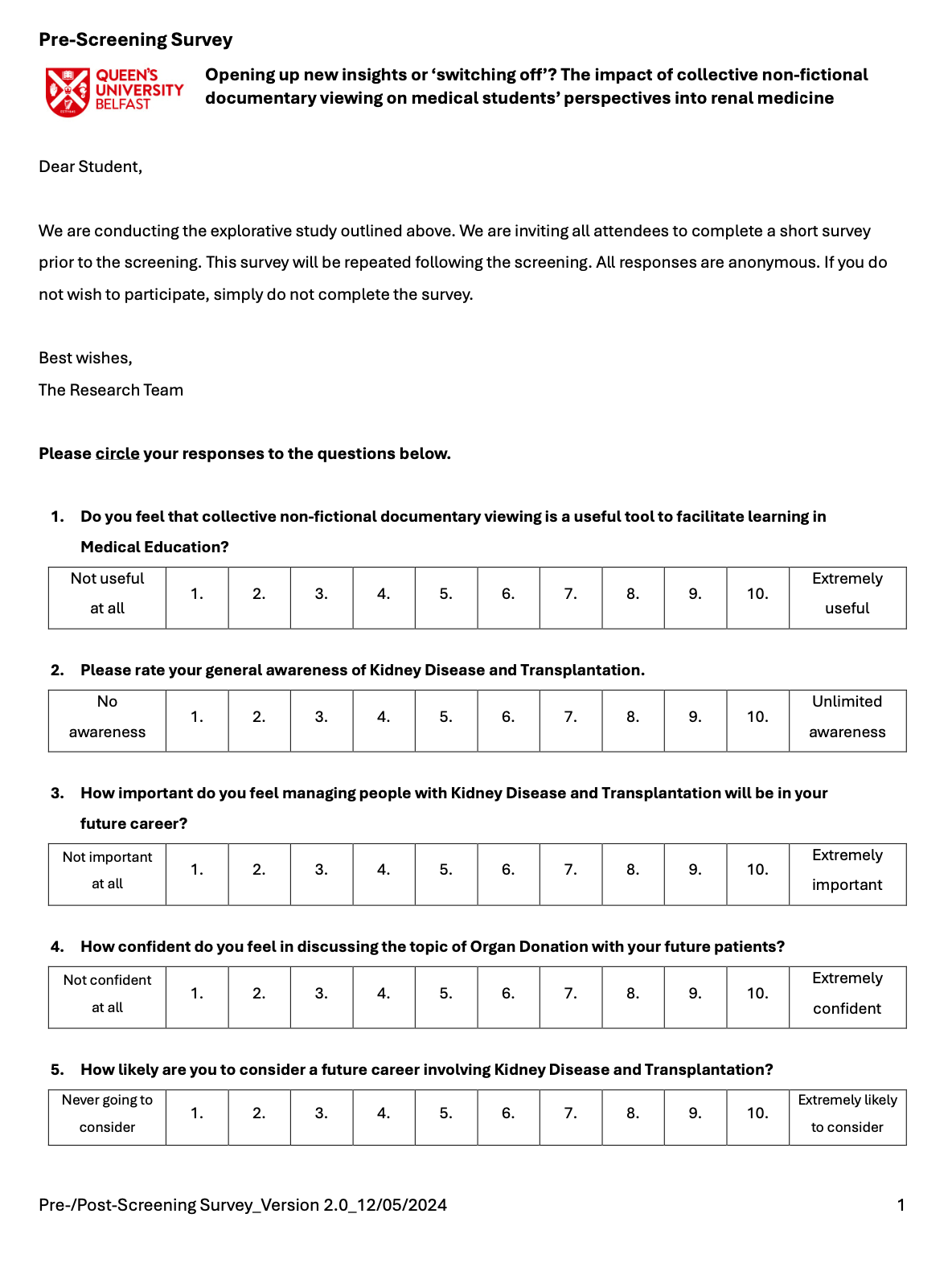


**Appendix 1b – post-screening questionnaire**


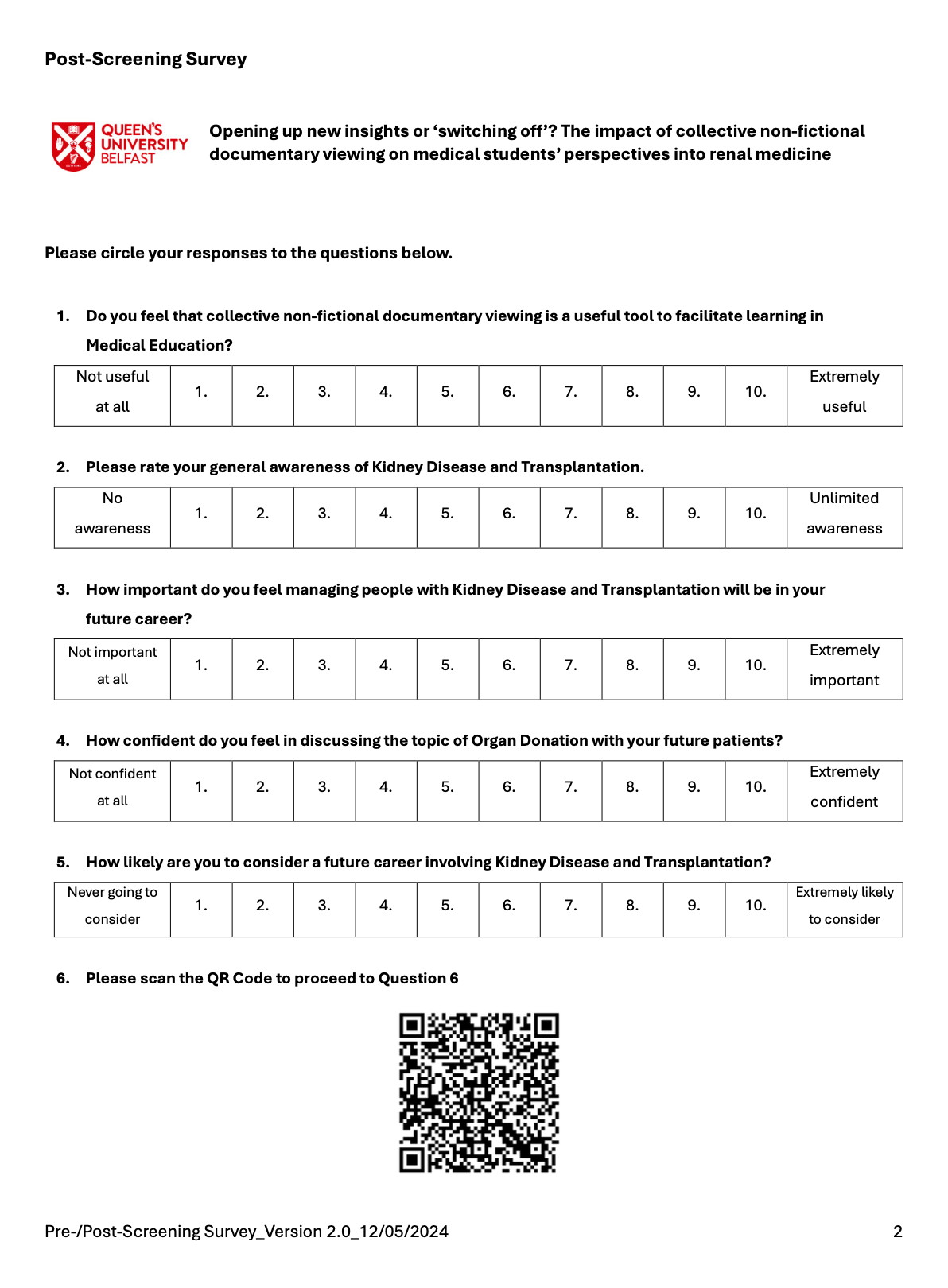

Supplement: online supplemental file 1 [file bmjopen-15-9-s001.docx]
