# Supplementary material for: Transformative learning through communal documentary viewing: a mixed methods study on kidney transplantation and organ donation in medical education
Source: BMJ Open. 2025 Sep 28;15(9):e095404. doi: 10.1136/bmjopen-2024-095404 (PMC12481357; doi:10.1136/bmjopen-2024-095404)
Supplement: Supplementary file 2 [file bmjopen-2024-095404.reviewer_comments.pdf]

PEER REVIEW HISTORY

BMJ Open publishes all reviews undertaken for accepted manuscripts. Reviewers are asked to complete a checklist review form and are provided with free text boxes to elaborate on their assessment. These free text comments are reproduced below.

ARTICLE DETAILS

Title (Provisional)

Transformative learning through communal documentary viewing: A mixed methods study on kidney transplantation and organ donation in medical education.

Authors

McCaffrey, Davog; Corr, Michael; Fergie, Ruth; Courtney, Aisling; Brown, Tim; Gormley, Gerard

---

VERSION 1 - REVIEW

Reviewer 1

Name Jowsey, Tanisha

Affiliation University of Auckland, Centre for Medical and Health Sciences Education

Date 19-Nov-2024

COI None

This is a fantastic paper. The team have designed an interesting and purpose driven arts-engaged initiative. They have partnered with the transplant community and followed a massive project - creation of a documentary about transplantation - with another massive project - evaluating whether the documentary made a difference to student learning and student thoughts about their future career options. There is solid engagement with appropriate theory, strong demonstration of sound qualitative knowledge, and I like the five targeted questions.

As if that's not enough, the authors then went on to run four focus groups, whereby they explored what students actually learned in more detail.

I wondered whether year 1 is the most appropriate year for this kind of educational intervention and I think it is. Career intentions often change a lot, as the authors will know, between year 1 and 5. Probably this should get a mention somewhere in the discussion or limitations. I'll leave that up to the authors.

Wishing the authors all the best with this, an inspiring piece of work. Thanks.

**Reviewer**	**2**
**Name**	**Owen, Katherine**
**Affiliation**	**Warwick Medical School**
**Date**	**24-Jun-2025**
**COI**	**None**

This is a very interesting paper which I enjoyed reading. The grounding in TLT was very successful and the qualitative elements were particularly compelling.

I would suggest reviewing the title of the paper as it gives no insight into the topic of the research.

VERSION 1 - AUTHOR RESPONSE

**Reviewer 1 Comments**		
This is a fantastic paper. The team have designed an interesting and purpose driven arts-engaged initiative. They have partnered with the transplant community and followed a massive project - creation of a documentary about transplantation - with another massive project - evaluating whether the documentary made a difference to student learning and student thoughts about their future career options. There is solid engagement with appropriate theory, strong demonstration of sound qualitative knowledge, and I like the five targeted questions.<br><br>As if that's not enough, the authors then went on to run four focus groups, whereby they explored what students actually learned in more detail.	We truly appreciate these supportive comments. We are heartened by the reviewers understanding of our project and its intended outcomes. We are pleased that the reviewer recognises the benefits of our mixed-methods approach, grounded in Transformative Learning Theory. Thank you!	N/A
I wondered whether year 1 is the most appropriate year for this kind of educational intervention and I think it is. Career intentions often change a lot, as the authors will know, between year 1 and 5. Probably this should get a mention somewhere in the discussion or limitations. I'll leave that up to the authors.	Thank you for this inspired comment. We have explicitly incorporated this point into the limitations section of our paper.	511-512

Wishing the authors all the best with this, an inspiring piece of work. Thanks.	The authors greatly value this positive comment and belief in our manuscript.	N/A
**Reviewer 2 Comments**		
This is a very interesting paper which I enjoyed reading. The grounding in TLT was very successful and the qualitative elements were particularly compelling.	We thank the reviewer for these supportive comments. We are encouraged that the reviewer recognises the compelling nature of the qualitative element of the study which was grounded in Transformative Learning Theory.	N/A
I would suggest reviewing the title of the paper as it gives no insight into the topic of the research.	Thank you for this comment. We have reflected on your comment and reviewed the title of the paper to offer greater insight into the research topic. The paper is now entitled, *"Transformative learning through communal documentary viewing:* A mixed methods study on kidney transplantation and organ donation in medical education".	
